# Melt Electrowriting of Complex 3D Anatomically Relevant Scaffolds

**DOI:** 10.3389/fbioe.2020.00793

**Published:** 2020-07-24

**Authors:** Navid T. Saidy, Tara Shabab, Onur Bas, Diana M. Rojas-González, Matthias Menne, Tim Henry, Dietmar W. Hutmacher, Petra Mela, Elena M. De-Juan-Pardo

**Affiliations:** ^1^Centre in Regenerative Medicine, Institute of Health and Biomedical Innovation (IHBI), Queensland University of Technology (QUT), Kelvin Grove, QLD, Australia; ^2^School of Dentistry, The University of Queensland, Herston, QLD, Australia; ^3^ARC ITTC in Additive Biomanufacturing, Queensland University of Technology, Musk Avenue, Brisbane, QLD, Australia; ^4^Medical Materials and Implants, Department of Mechanical Engineering, Technical University of Munich, Garching, Germany; ^5^Department of Cardiovascular Engineering, Institute of Applied Medical Engineering, Helmholtz Institute, Medical Faculty, RWTH Aachen University, Aachen, Germany; ^6^Institute for Advanced Study, Technical University of Munich, Garching, Germany; ^7^Department of Biohybrid and Medical Textiles (BioTex), AME-Institute of Applied Medical Engineering, Helmholtz Institute, RWTH Aachen University, Aachen, Germany; ^8^Translational 3d Printing Laboratory for Advanced Tissue Engineering (T3mPLATE), Harry Perkins Institute of Medical Research, QEII Medical Centre, Nedlands and Centre for Medical Research, The University of Western Australia, Perth, WA, Australia; ^9^Department of Mechanical Engineering, School of Engineering, The University of Western Australia, Perth, WA, Australia

**Keywords:** melt electrowriting, 3D printing, biomimetic, fused deposition modeling, personalized scaffolds

## Abstract

The manufacture of fibrous scaffolds with tailored micrometric features and anatomically relevant three-dimensional (3D) geometries for soft tissue engineering applications remains a great challenge. Melt electrowriting (MEW) is an advanced additive manufacturing technique capable of depositing predefined micrometric fibers. However, it has been so far inherently limited to simple planar and tubular scaffold geometries because of the need to avoid polymer jet instabilities. In this work, we surmount the technical boundaries of MEW to enable the manufacture of complex fibrous scaffolds with simultaneous controlled micrometric and patient-specific anatomic features. As an example of complex geometry, aortic root scaffolds featuring the sinuses of Valsalva were realized. By modeling the electric field strength associated with the MEW process for these constructs, we found that the combination of a conductive core mandrel with a non-conductive 3D printed model reproducing the complex geometry minimized the variability of the electric field thus enabling the accurate deposition of fibers. We validated these findings experimentally and leveraged the micrometric resolution of MEW to fabricate unprecedented fibrous aortic root scaffolds with anatomically relevant shapes and biomimetic microstructures and mechanical properties. Furthermore, we demonstrated the fabrication of patient-specific aortic root constructs from the 3D reconstruction of computed tomography clinical data.

## Introduction

Tissue engineering (TE) aims to develop methods of regenerating damaged tissues by combining cells and highly porous scaffolds to create biological substitutes that are capable of growth, remodeling and repair. Scaffolds aim to provide the 3D macro-geometry and micro-environment to guide cellular behavior toward the formation of a tissue, biologically and mechanically equivalent to the healthy native one (Hutmacher, 2001; O’Brien, 2011). To this end, the recapitulation of the fibrous nature of the ECM and the fiber architecture/alignment is crucial to achieve functional tissues (Ma, 2010). A number of techniques to fabricate fibrous scaffolds have been proposed for the bioengineering of soft tissues (Patterson et al., 2010; Capulli et al., 2016). However, the realization of a scaffold with controlled multiphasic microarchitecture and mechanical properties, and with anatomically relevant geometry and dimensions is still a challenge in biofabrication. Among the techniques to create fibrous scaffolds, melt electrowriting (MEW) has shown great potential because of its unique capability to deposit micron-sized fibers with ordered and pre-defined architectures. This advanced 3D printing technology combines principles of electrospinning and additive manufacturing (Brown et al., 2011; Muerza-Cascante et al., 2015; Bas et al., 2017; Wunner et al., 2017). In a typical MEW system, a molten polymer is extruded through a spinneret connected to a high voltage supply and deposited at the grounded collector, which is moving according to a predefined pattern. The net force between the surface tension and the attraction of the electrostatically charged droplet to the collector stabilizes the fluid column and prevents the jet from undergoing Raleigh Plateau instabilities (Stachewicz et al., 2017). The electric field plays a major role in MEW and governs important properties such as flight path of the polymeric jet and fiber diameter (Reneker et al., 2000). Once the printing conditions are established and maintained after an initial tuning phase, a robust printing process is achieved, however, changes in the electric field result in the breakage of the fluid column and accumulation of polymer at the spinneret with the consequent lack of control over fiber deposition. This determines the inability of MEW to print on complex 3D topographies that create continuous variations in the spinneret-to-collector distance. Thus, so far, melt electrowritten scaffolds have been exclusively fabricated on flat or cylindrical collectors, significantly restricting the potential of this technique for advanced tissue engineering and regenerative medicine applications.

The aim of this study was to overcome the technical boundaries of MEW in order to enable the manufacture of complex, anatomically relevant 3D fibrous scaffolds with tailored micrometric features for soft tissue engineering. Because of our interest in the cardiovascular field, we chose as an exemplary geometry the aortic root, as it features not only the irregular geometry of the sinuses of Valsalva (SV) but also a complex multiphasic histoarchitecture, including a unique tri-layered structure with a distinct collagen fiber distribution (Underwood, 2002; Tsamis et al., 2013). The aortic SV function as a supplementary component to the valve leaflets, by expanding a ring of flow vortices generated during systole, and promoting abrupt closure of leaflets (Toninato et al., 2016). Patients suffering from aortic root aneurysm or other valvular diseases present poor prognosis unless they receive root substitutes (Arabkhani et al., 2015). Inclusion and reconstruction of SV have been found instrumental in obtaining physiologically relevant structure-function relationship (Salica et al., 2016). Despite the long-recognized role of SV evident since the early 1990s (Robicsek, 1991), conventional aortic root replacements such as Dacron tubes do not include this important component leading to frequent long-term life-endangering complications (Robicsek and Thubrikar, 1999; De Paulis et al., 2001, 2002; Pisani et al., 2013). In addition, SV have been considered in very few tissue engineered heart valves where fine tuning fiber architecture and multiphasic properties across these constructs have been challenging (Lieshout et al., 2006; Nakayama et al., 2015; Capulli et al., 2017). Aortic root substitutes would ideally include not only the complex geometry of the SV but also their heterogeneous mechanical properties. However, the fabrication of such complex substitutes remains at best partial using current manufacturing techniques and requires further efforts toward root wall engineering to match the exquisite root properties (Butcher, 2018).

Here, we demonstrate that a two-component collector consisting of electrically conducting and non- conducting materials minimizes the electric field variations when printing a complex 3D geometry, thus allowing continuous controlled fiber deposition. Specifically, a metallic core enables the generation of the electric field, while a non-conductive polymeric shell reproduces the desired scaffold geometry without introducing variations in the electric field, which would otherwise result in jet instabilities. This solution does not require any modifications of existing set-ups and takes full advantage of the availability and versatility of 3D printing to realize any desired volumetric feature. We show that, by following this approach, we were able to produce real-size aortic roots with controlled fiber placement by MEW. We also demonstrate the capability to fabricate biomimetic tri-layered scaffolds mimicking the histoarchitecture and collagen fiber distribution of native aortic root and the potential of this technique for personalization and scalability.

## Materials and Methods

### Design of Aortic Root Model

The aortic root model design was guided by idealized dimensions suggested by Thubrikar (1990) whereby setting the basis for the computer aided (CAD) design of the aortic root 3D model. In Solidworks (Dassault Systèmes, Vélizy-Villacoublay, France), the model was designed comprising a 25 mm cylinder representing the aortic wall, with the addition of three sinus structures with dimensions listed in Supplementary Figure S1. A two-part model was developed representing the ascending aorta and sinuses (1) and left ventricular side (2) to allow for easy removal of the scaffold from the mandrel (Supplementary Figure S1). The aortic root model was scaled down to 20, 15, and 10 mm to fabricate models over a range of relevant anatomies, highlighting the scalability of this fabrication platform.

### Electric Field Simulation

The *in silico* electric field model was simulated in COSMOL Multiphysics software (version 5.3, COSMOL Inc., United States). The MEW setup was 3D modeled in Solidworks (Dassault Systèmes, Vélizy-Villacoublay, France) and imported to COSMOL along with the aortic root model. The electrical conductivity of the materials was defined according to the physical properties of materials established in the COSMOL Metaphysics (Aluminum mandrel: 3.774e7 [S/m], Titanium: 7.407e5 [S/m], Air: 5e-15 [S/m]) while the dielectric constant of 3D printed PLA was taken from previously published papers (die-electric constant:3.471) (Huber et al., 2016). The electric field strength (EFS) during the MEW process was modeled by defining the spinneret position at a collector distance of 10 mm from the wall for 10 kV, where voltage was kept constant for both mandrel models.

### 3D Printing of the PLA Aortic Root Model

The aortic root mold was fabricated via fused deposition modeling (FDM, WomBot, Keysborough, Australia) using poly-lactic acid (PLA, Bilby 3D, Australia) heated to 210–230°C. STL files of the aortic root model were imported into 3D Central (Simplified 3D, United States) to generate g-code with 0% infill, 6 perimeters, 0.2 mm layer height, 1200 mm/min. Following printing, the 3D printed models were smoothed using fine (320 grade) sand paper (Duramax, Australia) to minimize the surface roughness that could potentially damage the scaffolds. In the case of the patient-specific aortic root model, DICOM imaging slices collected from the CT scan of the patient’s aorta (Office of Research Ethics and Integrity of the Queensland University of Technology, approval number QUT1900000599) were converted to a STL file using InVesalius software (Center for Information Technology Renato Archer, São Paulo, Brazil) for further processing. STL files were then smoothen and transformed into a straight configuration using Netfabb software (Autodesk, California, United States) to allow for the integration of the Al mandrel. This finalized model was used for 3D printing the personalized scaffold using MEW. Lastly the Titanium aortic root model was manufactured.

### Scaffold Fabrication

Custom-made MEW Al-Ti and Al-PLA collectors were used to fabricate MEW scaffolds replicating the macroscopic geometry of aortic root including the sinuses of Valsalva. In this process, medical grade PCL pellets (Purasorb^®^ PC 12, Purac Biomaterials, Netherlands) are heated at 80 (syringe) and 92°C (spinneret) in a plastic syringe. 2.0 bar air pressure pushes the molten polymer through a 23 G needle where high voltage drags the fiber down onto a rotating mandrel collector while translating the mandrel along its axis. The spinneret was kept at 10, 12 mm for Al-Ti and 10 mm for Al-PLA from the walls, positioning it at 7.5 mm from the highest point of the sinuses. The voltage was set at 6, 8, and 10 kV for the Al-Ti model and 10 kV for the Al-PLA model while other MEW parameters were kept constant. All scaffold groups were fabricated for 7500 cycles of collector translation moving at 1000 mm/min keeping the fiber collection time constant regardless of the scaffold architecture. Scaffolds were sprayed with Ethanol 70% and slipped of the two-part PLA model specifically designed to ease removal of the scaffolds.

### Morphological Characterization

In-process MEW images were acquired using a handheld digital microscope (AM7115MZTL, Dino-Lite, Taiwan) and a handheld video camera recorder (HDR-PJ410, Sony, Tokyo, Japan) to monitor the MEW process when printing on the different mandrel models. Following printing and removal of scaffolds from the 3D printed model, macroscopic images were acquired with a microscope camera to illustrate the scaffold conformity to the model (Nikon DS-Fi2, Japan). For scanning electron microscopy (SEM), sections of aortic root scaffolds were taken from the walls and sinuses (*n* = 3) using an 8 mm biopsy punch (Kai medical, Australia) and mounted onto SEM stubs for gold sputter coating for 90 s. SEM images were taken from the inside of the scaffolds to ensure that potential roughness caused by the surface of the PLA mold could be detected. SEM images were acquired using a Mira3 SEM (TESCAN, Brno, Czechia) with 5 kV beam voltage, 200× magnification, 10.53 mm working distance. Fiber diameters were measured using SEM images using ImageJ and reported as average ± standard deviation for a total of *n* = 81 measurement points. Fiber diameter measurement was carried out for three independent complete scaffolds cut into three pieces (each containing a sinus) for each of three winding angle configurations. Nine measurements were taken from the wall and sinuses of each scaffold separately, to ensure fiber diameter consistency across every sample and different prints.

### Fibrin Preparation and Polymerization

Fibrinogen was prepared as described previously (Moreira et al., 2016). In short, dissolved lyophilized fibrinogen (Calbiochem) was dialyzed against tris-buffered saline (TBS; pH 7.4) for 12 h utilizing a 6000–8000 molecular weight cut-off membrane (Novodirect). The obtained fibrinogen solution was sterilized by filtration (0.2 μm pore size, Corning^®^), and the concentration was defined by measuring the absorbance at 280 nm using an Infinite M200 spectrophotometer (Tecan Group Ltd.). The fibrin gel components of MEW/fibrin composite (5.0 mL in total) consisted of 2.5 mL fibrinogen solution (10 mg/mL), and the fibrin polymerization starting solution composed of 1.75 mL TBS, 0.375 mL 50 mM CaCl-2 (Sigma) in TBS, and 0.375 mL 40 U/mL thrombin (Sigma). The components were introduced into a 3D printed (Objet Eden350) square shaped mold of 225 mm^2^ to composite the scaffolds for burst strength tests.

### Burst Strength Test

Burst strength of the MEW fibrin composite scaffolds was obtained using a custom-made device equipped with a pressure gauge (Jumo Midas pressure transmitter, JUMO GmbH & Co. KG) and peristaltic pump (IPC Ismatic, IDEX Heath & Science GmbH), reported elsewhere (Moreira et al., 2016). Squared samples from the aortic wall and sinuses with 1.5 cm^2^ area were mounted into the measuring device. Phosphate buffered saline (PSB) was pumped into the device with increasing pressure until structural failure occurred and the maximum pressure was measured. Burst strength samples were obtained either from the center of the sinuses or from the top of the root wall, and not on the overall scaffold.

### Suture Retention Test

To assess the suture retention strength, MEW fibrin composite scaffolds (*n* = 3) were tested using an Instron tensile tester with a 100 N load cell (Zwick Roell, Ulm, Germany) according to ISO 7198. Samples were mounted into a pneumatic clamp and 4-0 suture line was threaded through the sample at a depth of 3 mm below the proximal surface of the aortic root wall and clamped. The suture line was then stretched until the sample was torn indicating failure where the maximum load was recorded as the suture retention strength.

### Cell Seeding

Umbilical cords were kindly provided by the Department of Gynecology at the University Hospital Aachen in accordance with the human subjects’ approval of the ethics committee (EK 2067). Human umbilical vein smooth muscle cells (HUVSMCs) were isolated by stripping out the umbilical cord vein, removing the remaining adherent connective tissue, cutting 1 mm tissue rings, and placing them in cell culture flasks. Outgrowth of HUVSMCs from the tissue rings onto the tissue culture plastic (TCP) was observed after 1 to 2 weeks. HUVSMCs were cultured in Dulbecco’s modified Eagle medium (DMEM; Gibco) supplemented with 10% fetal calf serum (FCS; Gibco) in 5% CO2 and 95% humidity at 37°C up to a confluence of 80% to 90% and subsequently passaged. Cells in passage four were used for the seeding experiments.

Small pieces (1 cm × 1 cm) of MEW scaffolds from 60 degrees and tri-layered groups were cut from the scaffold root wall and used for the cell seeding experiments. MEW scaffolds were sterilized by dipping in 70% ethanol followed by evaporation inside the biosafety cabinet. After being completely dried, the scaffolds were washed with PBS and clamped into 48-well plate inserts (CellCrown, Scaffdex). HUVSMCs were enzymatically detached from the TCP by 0.25% trypsin/0.02% ethylenediaminetetraacetic acid (EDTA) solution (Gibco), collected in a conical tube (Sarstedt) and counted using a Neubauer chamber. Cells were centrifuged at 500 × *g* for 5 min and resuspended in cell culture medium at a concentration of 10 million cells per mL medium. The inserts were placed inside a 24-well plate (VWR international, Pennsylvania, United States) in a standing position with the scaffold facing upward. A direct seeding was performed by slowly pipetting 100 μL of cell suspension onto the surface of the MEW scaffolds. The constructs were incubated for 2 h at 37°C, 5% CO_2_ and 95% humidity. After this, the cell crowns were laid onto their side and medium was added to the wells until the seeded surface was completely covered. The culture was maintained for 72 h.

### Immunohistochemistry

To perform immunohistochemical analysis of the cell-seeded scaffolds, samples were fixed in methanol-free 4% PFA in PBS for 1 h at room temperature and washed with PBS afterward. Cell membranes were permeabilized using 0.1% Triton-PBS for 1 h at room temperature. Samples were incubated with 100 nM working solution of fluorescent phalloidin (Acti-stain^TM^ 488; Cytoskeleton, Inc.) for 1 h at 37°C, this was followed by a washing step with PBS and a nuclei counterstaining with DAPI (Molecular Probes). The stained samples were visualized using a Two photon laser scanning microscopy (TPLSM) setup consisting of an Olympus FluoView 1000MPE with a 25× water objective (NA 1.05, Olympus, Tokyo, Japan), a mode-locked MaiTai DeepSee Titanium-Sapphire Laser (Spectra-Physics, Stahnsdorf, Germany), and the FluoView FV10 4.2 acquisition software.

### Micro-Computed Tomography

Disk shaped samples with 8mm diameter were biopsy punched from wall and sinuses of all scaffold groups and transferred into 9 mm μCT tubes. Microtomography (microCT) was carried out to provide information on the scaffold 3D morphology (microCT50, Scanco Medical, Bruettisellen, Switzerland – 9 mm tube, 3 μm voxel size, 45 kV, 200 μA). The lower threshold was set at 50 and the upper threshold was set at 1000 to isolate the PCL scaffold from the background for the evaluation of different morphological parameters. Scaffold fiber volume was evaluated by microCT software (Scanco Medical, Bruettisellen, Switzerland). Scaffold thickness was measured for every sample by first converting the CT scan files to DICOM format and calculating the number of stacked layers at five points of every selected sample using the free software package distribution Fiji, based on ImageJ v1.52c (National Institute of Health, United States). Accordingly, multiplying the number stacked layers and the voxel size of the micro CT machine (3 μm) determined the overall scaffold thickness. Finally, scaffold porosity was calculated from the scaffold fiber volume and true overall volume of an 8 mm disc, to ensure that the variability of thickness for every sample is considered.

### Statistics

In order to compare the fiber diameter and winding angle, *n* = 81 points of measurement were taken from wall and sinuses from *n* = 3 scaffolds for every scaffold architecture and a one−way ANOVA test followed by a Tukey multiple comparison test was utilized to assess statistical significance (GraphPad, Prism 7). The asterisks above every candlestick column illustrate statistical significance with respect to all other columns, unless specified otherwise. Burst strength of scaffolds with both single layer and tri-layered architecture scaffolds were reported as mean ± standard deviation for *n* = 3 samples representing the wall and sinuses where one−way ANOVA test followed by Sidak’s multiple comparison test was performed to control the familywise error rate. In addition, an unpaired *t*-test was performed for investigating the burst strength difference between wall and sinuses of the tri-layered scaffolds and the suture retention strength difference between 60° and tri-layered scaffolds. Lastly, fiber volume, thickness and porosity for every scaffold group was calculated *n* = 3 where these values are reported as mean ± standard deviation. A one−way ANOVA with Sidak’s multiple comparison *post hoc* component was also used to test the statistical significance of this set of data. Values of *p* < 0.05 were considered significant. The level of significance was indicated with asterisks as follows: (*p* < 0.0001^****^, 0.0001 < *p* < 0.001^∗∗∗^, 0.001 < *p* < 0.01^∗∗^, 0.01 < *p* < 0.05^∗^.

## Results and Discussion

### Fabrication of Anatomically Relevant Aortic Root Scaffolds

**FIGURE 1:**
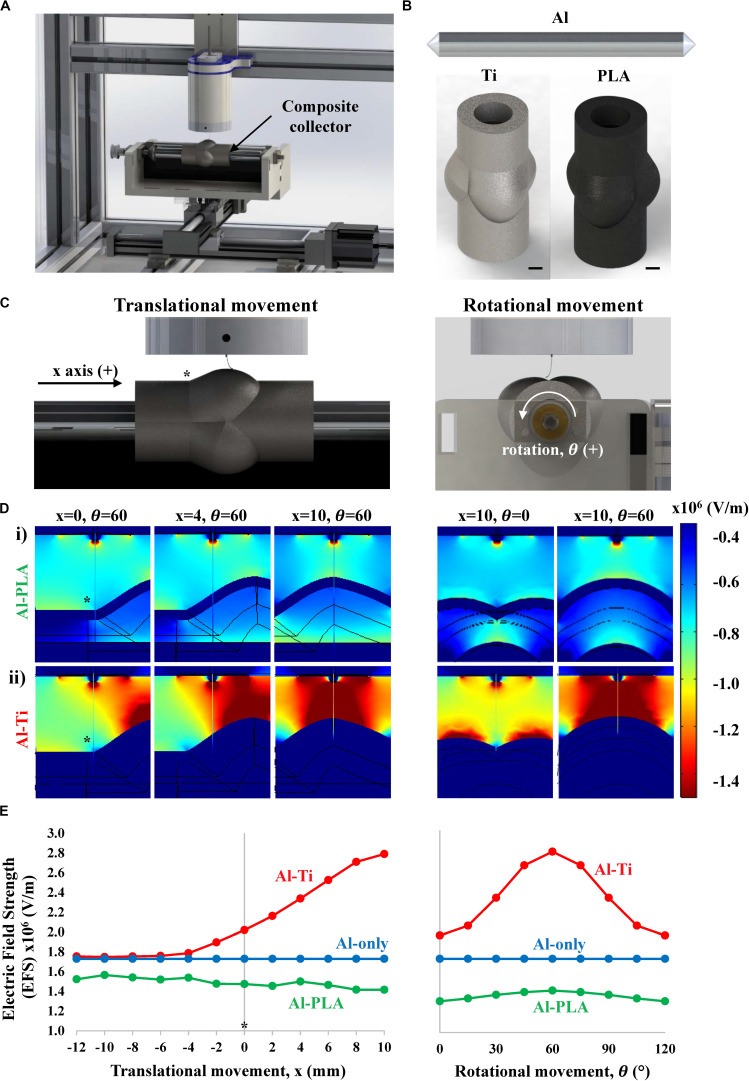
Simulation of the electric field strength (EFS) (V/m) established between the spinneret and aortic root model during MEW. **(A)** Schematic representation of the mounted collector onto a standard MEW device. **(B)** Aluminum only (Al-only), Al-titanium (Al-Ti) and Al-polylactic acid (Al-PLA) mandrel models used for electric field simulation (scale bar = 5 mm). **(C)** Front (left) and side (right) plane views of the MEW setup used in this study, * indicates the point on the mandrel where *x* = 0 **(D)** Heat maps showing the EFS distribution when printing on i) Al-PLA and ii) Al-Ti collectors at different stages of the translational (left) and rotational (right) movements, **(E)** Quantitative EFS simulation results when printing on the different double component collectors: Al-Ti (red), Al-PLA (green) and Al-only (blue).

To maintain a stable polymer jet and, consequently, a reproducible printing process during MEW, it is essential to preserve a constant electric field (Wunner et al., 2018). Fast changes of working distance associated with 3D geometry of metallic collectors would cause electric field variabilities and consequent breakage of the fluid column, introducing printing defects. Here we hypothesized that a stable jet could be achieved by using a hybrid collector instead of a conventionally employed metallic one, as schematically shown in Figures 1A,B. Specifically, a conductive cylindrical Al mandrel would enable the formation of a constant electric field while a coaxial non-conductive PLA mold would collect the fibers into the desired macroscopic scaffold geometry without causing significant field variations (Al-PLA), thus avoiding the disruption of the printing process. Initially, computational simulations of the electric field strength (EFS) associated with the Al-PLA collector were performed. For comparison, two additional combinations of mandrel-mold collectors were also examined: Al mandrel combined with a coaxial conductive Ti mold (Al-Ti, fully conductive), and Al mandrel only (Al-only) (Figures 1A,B). The aortic root geometry was obtained by 3D printing based on literature as explained in Supplementary Figure S1 (Thubrikar, 1990). Within a complete printing cycle, only small changes in the EFS associated to the translational (Figure 1C, left column) and rotational (Figure 1C, right column) movements were predicted for the Al-PLA collector (Figure 1D, Al-PLA) (Supplementary Video 1 and Supplementary Video 2). This was in strong contrast with the EFS heterogeneities anticipated for the full metallic collector as shown in Figure 1D, Al-Ti and Supplementary Videos 3, 4. Quantitative assessment of EFS using this *in silico* model predicts a 45% rise in EFS as the spinneret moves toward the sinuses of the fully conductive collector (Al-Ti) during both translation and rotational movement, whereas this increase was not observed for the Al-PLA model (Figure 1E). Given the dielectric breakdown strength of air (3 × 10^6^ V/m) and the humidity conditions (30–40%), we speculated that this significant increase in EFS could result in dielectric breakdown when printing on the Al-Ti mandrel. The Al cylindrical mandrel was modeled as a control group, which resulted in a stable field, confirming the capability of MEW to print plain cylindrical scaffolds.

Next, we fabricated aortic root scaffolds via MEW using collectors with the same geometry and materials as the ones used in the EFS model. The core conductive Al mandrel was fabricated by classical machining, whereas the molds featuring the SV were 3D printed in PLA and Ti. Once assembled, the double component collectors were easily coupled to the motorized stage without requiring any modifications of the existing set-up. The stability of the MEW process was first assessed by visual inspection of the jet and of the resulting printed samples, and subsequently further evaluated at the micrometric scale by scanning electron microscopy (SEM).

**FIGURE 2:**
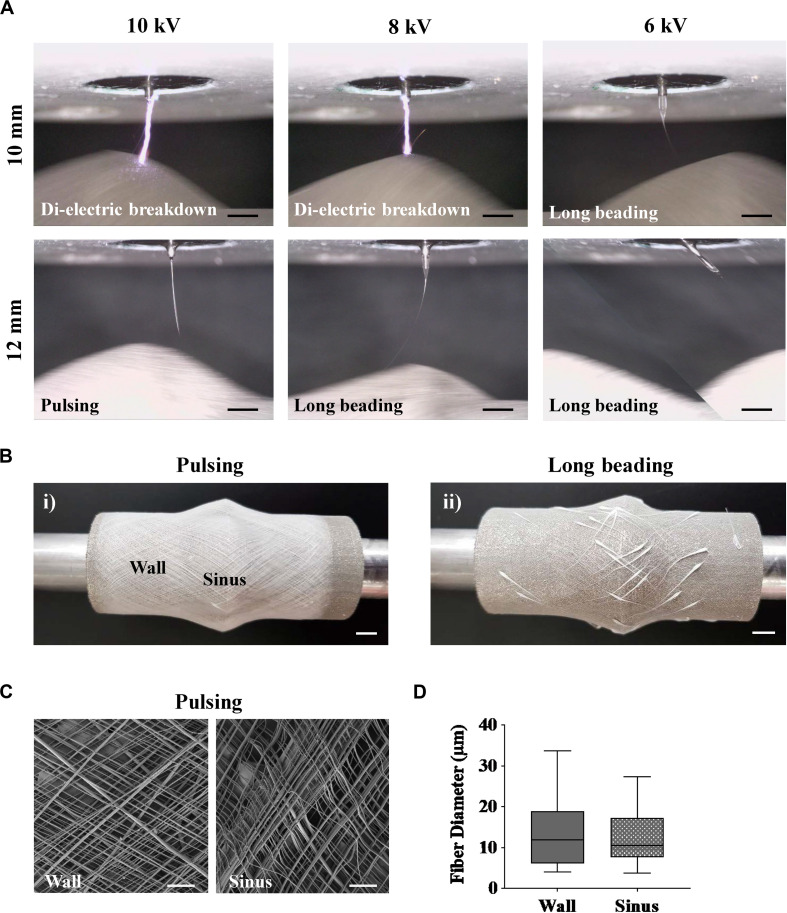
MEW performed on the fully conductive Al-Ti. Taylor cone formation and fiber flight path were investigated as a function of collector-to-spinneret distance and applied voltage, while pressure, temperatures and speeds were kept constant. **(A)** Time lapse images of MEW process using an Al-Ti collector. **(B)** Representative images of scaffolds printed with (i) pulsing and (ii) long beading phenomena on the Al-Ti collector. **(C)** Representative SEM images of a scaffold successfully fabricated on the Al-Ti collector at 12 mm working distance and 10 kV. **(D)** Morphological analysis of scaffolds fabricated using the Al-Ti collector. (*n* = 3, *n* = 81 measurement points). (Scale bars: **(A,C)** = 2 mm; **(B)** = 5 mm).

MEW performed on the Al-Ti collector resulted in unstable printing conditions, which were in agreement with the field inhomogeneity predicted by the simulations. In particular, for the 10 mm spinneret-to-collector distance, voltages equal or greater than 8 kV were sufficient to cause dielectric breakdown, confirming the prediction made using the *in silico* model of EFS (Figure 2A and Supplementary Videos 5, 6). A reduction of the voltage to 6 kV was enough to prevent the dielectric breakdown, but not to circumvent other undesirable events associated with unstable printing conditions, which were also detected at higher voltages and larger working distances (8 kV, 12 mm) (Supplementary Videos 7, 8). Specifically, accumulation of the polymer melt without the formation of a Taylor cone was observed at the spinneret when the applied voltage was too low (6 kV), and this caused a large mass of polymer melt to be dragged onto the collector, creating long beading defects (Figures 2A,B) (Supplementary Video 9) (Hochleitner et al., 2016). Printing at 12 mm collector-to-spinneret distance and 10 kV allowed to maintain a Taylor cone and a continuous flow jet without any visible dielectric breakdowns, permitting the fabrication of complete scaffolds (Supplementary Video 10) (Hochleitner et al., 2016). However, further analysis by SEM discovered substantial variability of the fiber diameters both on the wall and sinuses, which was a clear indication of fiber pulsing (Figure 2). Fiber pulsing is a common phenomenon that occurs as a result of imbalance of forces during MEW process, leading to inconsistencies in the extruded mass flow of polymer and consequently to inhomogeneous fiber diameters (Hochleitner et al., 2016). The occurrence of long bead defects and fiber pulsing renders the process uncontrollable and irreproducible.

In contrast, the Al-PLA collector facilitated the formation of a proper Taylor cone and regular fiber flight path resulting in a robust printing process at the same printing conditions that caused dielectric breakdown for the Al-Ti collector (optimal conditions found at 10 mm and 10 kV). In general, closer distances are sought in order to reach a better agreement between the programmed and the real fiber lay-down pattern. Snapshots of the fabrication process for both the Al-Ti and Al-PLA model were taken every 30 s to better capture the stability of the polymer jet when printing on the Al-PLA model (Supplementary Figure S2).

Previous studies had used a double-component collector in solution electrospinning for the manufacture of anatomically shaped heart valve scaffolds (D’Amore et al., 2018; Coyan et al., 2020). Contrarily to our concept and implementation, in these studies the collector surface was composed of two materials with different conductivity (as opposed to our conductive core and anatomically shaped non-conductive shell approach), to spatially control fiber deposition and avoid fiber accumulation in specific regions, which would affect the valve functionality.

**FIGURE 3:**
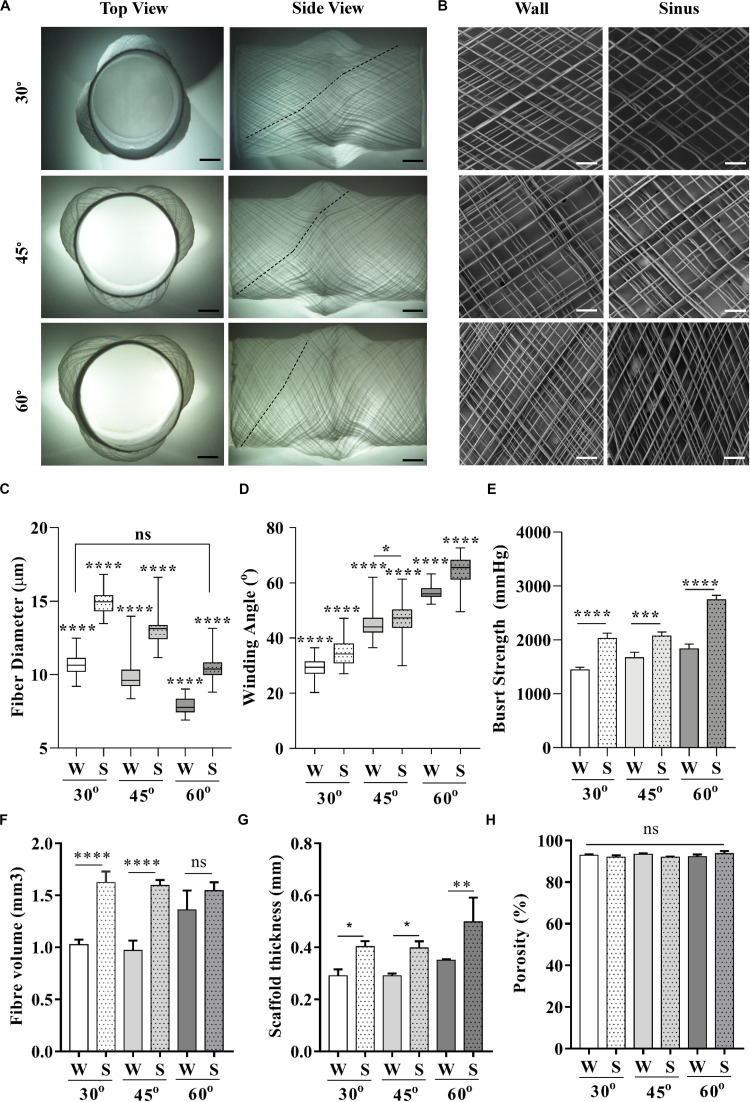
Characterization of scaffolds printed at 30, 45, and 60° winding angle. **(A)** Macroscopic top and side view pictures of full scaffolds, **(B)** SEM images taken individually from the wall and sinuses. **(C)** Fiber diameter and **(D)** winding angle measurements on the wall and sinuses across all groups. **(E)** Burst strength of scaffolds with varying winding angles composited with fibrin hydrogel (*p* < 0.05 were considered significant). (Scale bars: **(A)** = 5 mm, **(B)** = 2 mm); (statistical analysis: **(C,D)** = One way ANOVA with Tukey multiple comparison; **(E–H)** = One way ANOVA with Sidak multiple comparison; *p* < 0.0001****, 0.0001 < *p* < 0.001***, 0.001 < *p* < 0.01**, 0.01 < *p* < 0.05*).

Aortic root scaffolds were fabricated on the Al-PLA collector by keeping a constant translational speed of 1000 mm/min and varying the mandrel’s rotational speed to obtain three different winding angles (Brown et al., 2012). All scaffolds conformed to the shape of the 3D printed PLA aortic root model (Figure 3A), with no apparent defects. We were able to obtain winding angles of 30, 45, and 60° on the scaffold wall, as estimated by analysis of SEM images (Figures 3B,C), by setting the rotational speed to 6.4, 11.0, and 19.1 rpm, respectively. For all scaffolds, fibers deposited on the sinuses presented higher winding angles than those deposited on the wall, which can be explained by the increased tangential speed as a result of the larger mandrel diameter at the SV. The direct relationship between tangential speed and winding angle observed in this experiment agrees with a study conducted by Jungst et al. (2015) on a simple non-complex mandrel.

Morphological analysis of these scaffolds by SEM revealed minimal variability of fiber diameters across the entire scaffold surface (Figure 3D) compared to those obtained with the Al-Ti collector (Figure 2D). A slight but significant reduction in fiber diameter caused by thinning of the polymer jet with increasing rotational speed (Figure 3C) was observed. Additionally, fibers were significantly thicker over the sinuses than on the wall, independent of the winding angle. This can be explained by the smaller thinning effect on the fibers associated to shorter spinneret-to-collector distances (Figure 3C). While regional heterogeneities in fiber diameter and winding angle can be beneficial for some applications, as we will next show for the aortic root scaffolds, these microarchitectural properties can be tuned and maintained by altering the rotational speed for applications that requires a homogeneous fiber structure, as shown in Supplementary Figure S3.

The burst strength of the scaffolds was measured to assess the effect of heterogeneous fiber diameters and winding angles across wall and sinuses on the mechanical properties. To this end, we composited the scaffolds with fibrin as an exemplary hydrogel. Fibrin is commonly used as cell carrier in tissue engineering applications, including cardiovascular ones (Jockenhoevel et al., 2001; Moreira et al., 2015, 2016; Toosi Saidy et al., 2019). Results showed a significant increase of burst strength on the sinuses compared to the walls for all scaffold groups (Figure 3). Although to the best of our knowledge, there are no data in the literature on the difference of burst strength between the wall and SV, higher tensile stiffness of SV (Azadani et al., 2012) suggests a higher burst strength according to a number of studies that correlate tensile stiffness to burst strength (Laterreur et al., 2014; Geelhoed et al., 2019). This is in agreement with our burst strength results presented for the wall and SV of MEW/fibrin scaffolds.

To further investigate the importance of other contributing factors such as fiber volume, sample thickness, and porosity toward this effect, we performed micro-computer tomography (μCT) analysis on 8 mm disk-shaped biopsies of both regions for all scaffold groups. Interestingly, μCT analysis revealed larger sample thickness and fiber volume over the sinuses across all samples. This could be explained based on the constant rotational speed and printing time kept for each of the scaffold groups, which caused higher fiber volume to be collected over the regions exposed to the fastest tangential speed, i.e., the sinuses. Enhanced fiber volume combined with increased fiber diameter over the sinuses directly contributed to larger sample thickness. The combination of increased sample thickness and fiber volume would suggest an increase of burst strength on the sinuses; however, the influence of larger fiber volumes over the sinuses superseded the contribution of fiber diameter to the overall thickness and total fiber volume collected. Intriguingly, there was no significant difference in terms of porosity among regions and scaffold groups (Figure 3F), which suggests that the fiber volume is the main contributing factor toward burst strength. These experiments confirmed the predicted *in silico* results suggesting that the quasi-uniform EFS distribution for the hybrid Al-PLA collector would be appropriate to achieve stable MEW printing conditions with controlled fiber deposition, thus validating the initial hypothesis.

There are other possible strategies that could be applied to keep the electric field constant during MEW of complex geometries. One of them would be to motorize the printer head and synchronize its vertical movement with the translation and rotation of the collector to keep the spinneret-to-collector distance constant. Altering the applied voltage during the printing process with a programmed module would be another approach to control the electric field during the course of MEW process. Recently, we demonstrated that the combination of both, a motorized print head and controlled incremental increase in applied voltage, safeguards a stable jet during the printing process of flat scaffolds, enabling to reach up to 7 mm scaffold thickness (Wunner et al., 2018). However, following a similar approach on a geometrical complex mandrel would require substantial additional programming and in-process control to adjust the voltage as the mandrel is rotating and translating. In addition, this requires fundamental understanding of electrostatic interactions and its influence on the forces imparted on the polymeric jet to predict the required voltage that retains these forces at different spinneret to collector distance, which is highly challenging. Another strategy would be to upgrade the MEW printer with the installation of a robotic arm to ensure a perpendicular and constant spinneret-to-collector distance. This approach, however, would require significant adjustments of both hardware and software to allow for the complex synchronization of the movements. Our proposed double component collector approach, on the contrary, presents a simple, cost-effective and robust method, which takes full advantage of the availability and versatility of 3D printing to realize any desired geometry.

### Fabrication of Biomimetic Tri-Layered Aortic Root Scaffolds

**FIGURE 4:**
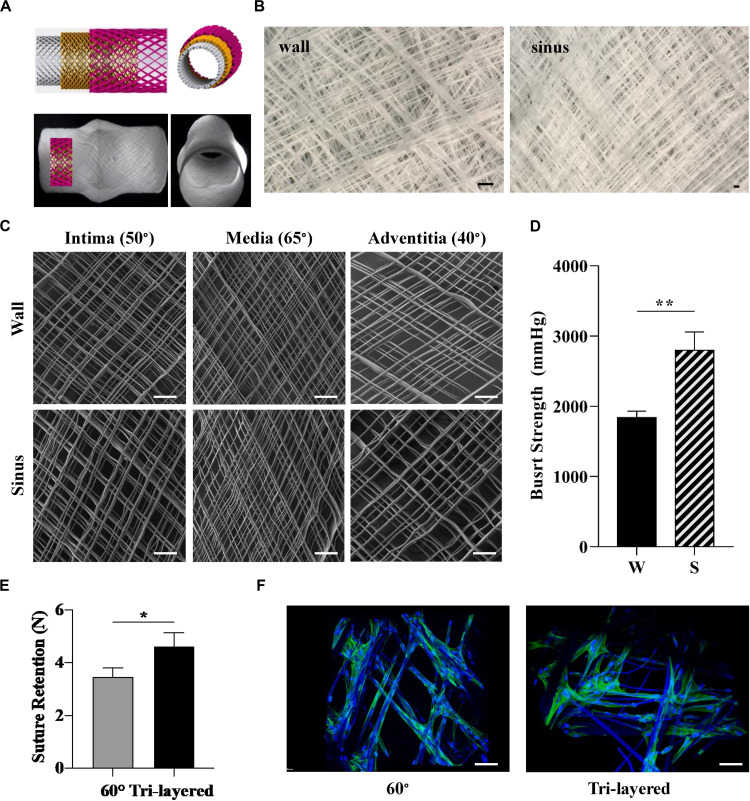
Design and fabrication of biomimetic tri-layered aortic root scaffolds. **(A)** 3D illustration of the fibers at the defined angles across the three layers (top) and representative images of the scaffold (bottom). **(B)** Stereomicroscope images of tri-layered scaffold at the wall (left) and sinus (right). **(C)** SEM images of each of the individual layers forming the biomimetic scaffold: intima (50°), media (65°) and adventitia (40°) taken from the wall and sinus. **(D)** Burst strength of tri-layered scaffold measured at the wall and sinus. **(E)** Suture retention of tri-layered scaffold compared to scaffold with 60° winding angle, which presented similar burst strength. **(F)** Attachment and alignment of HUVSMC after 4 days of culture on scaffolds with 60° winding angle and tri-layered, measured by two-photon microscopy. (Scale bars: **(B,C,F)** = 200 μm); (statistical analysis: Unpaired *t*-test; 0.001 < *p* < 0.01**, 0.01 < *p* < 0.05*).

Collagen fibers present a distinct and varying directionality within the tri-layered wall of native aortic roots (Schriefl et al., 2012). Given the influence of fiber arrangement and orientation on tissue mechanical properties, we manufactured tri-layered, anatomically relevant scaffolds mimicking the native collagen fiber orientation. Our design was guided by studies that have focused on microscopy and numerical modeling of collagen fibers in individual layers of the human coronary artery with fibers oriented at 50° in the intima, 65° in the media and 40° in the adventitia with respect to the central axis of the aorta (Figure 4A; Schriefl et al., 2012). The translational and rotational speeds were calculated accordingly to mimic these orientations in three superposed layers (Supplementary Figure S3) which were printed in a single print (Figure 4B). The morphological characterization of each individual layer by SEM confirmed the targeted winding angles (Figure 4C). While biomimetic design strategies based on the blueprint of the native tissue are highly desired, fabricating bioinspired multi-layered and multiphasic structures has been a major challenge due to the limitations associated with currently available fabrication technologies (Ma, 2010; Wang et al., 2016). Techniques such as solution electrospinning (Hasan et al., 2014; Liu et al., 2017; Akentjew et al., 2019) and jet spinning (Capulli et al., 2017) have been used to produce scaffolds with aligned fibers. However, predefining the angle of deposited fibers and fabrication of complex shaped scaffolds that mimic the individual layers of an aorta and includes the SV remains unexplored. The proposed MEW setup for the first time allows for the fabrication of complex shaped aortic root scaffold including SV with tri-layered fiber architecture mimicking that of a native coronary artery. The tunability of fiber deposition angle and alignment presented in Figure 3 as a single architecture scaffold and in Figure 4 as a tri-layered scaffold opens up avenues for the fabrication of scaffold for other soft tissues with hierarchical and heterogeneous collagen fiber architecture.

The tri-layered scaffolds composited with fibrin revealed a significant difference in burst strength between the sinus and the wall (Figure 4D), similarly to the scaffolds produced with just one winding angle (Figure 4E).

Suture retention is an important property for the implantability of the scaffolds (Pensalfini et al., 2018). Therefore, we next investigated the suture retention strength of the tri-layered scaffold and compared it to the 60° winding angle scaffold, as it presented a similar burst strength but a different fiber arrangement. The tri-layered scaffold displayed a significantly higher suture retention strength compared to the 60° scaffold (Figure 4E). Given that the printing time and number of printing loops were kept constant for both group of scaffolds, this is potentially associated with the difference in fiber arrangement.

To assess the potential of this biomimetic design approach for vascular tissue engineering, burst strength and suture retention of the tri-layered scaffolds where then compared to polytetrafluoroethylene (ePTFE) grafts, the gold-standard synthetic vascular grafts used in the clinic. Importantly, burst strength on both the wall and sinuses of the tri-layered MEW/fibrin scaffolds captured the reported values of ePTFE small caliber grafts (1323 ± 383 mmHg) generally used for aorta reconstruction (Kim et al., 2011), which is well above the pressure experienced by native aortic root under patho-physiological conditions (90–200 mmHg) (Rothwell et al., 2010). Moreover, MEW/fibrin scaffolds presented burst strength similar to that of the bovine arteries, native saphenous vein and internal mammary artery (Konig et al., 2009). The heterogeneity of burst strengths obtained for the tri-layered composite scaffold better captures the behavior of native aortic roots than the synthetic grafts that do not include SV. The suture retention strength of the tri-layered composite scaffold was also significantly higher than that of the ePTFE (3.19 ± 1.49 N) (Kim et al., 2011), further highlighting its potential suitability for surgical implantation. High suture strength is also beneficial for the incorporation of aortic valve scaffolds in the root, given the importance of this property to enable successful integration. Scaffolds specifically designed for the aortic root with great suture retention and burst strength are also essential for heart valve tissue engineering given the growing body of literature on the importance of heterogeneous scaffolds specifically mimicking architecture and mechanical properties of aortic root and valve (Butcher, 2018).

Overall, the tri-layered scaffold not only performed comparably well in terms of suture retention and burst strength to the gold standard grafts but also incorporated the SV and heterogeneous fiber architecture across the wall and sinuses, both important characteristics of the native root that are not considered in the design of available grafts. To the best of our knowledge, this is the first report on the fabrication of a tri-layered aortic root fibrous scaffold that includes the SV.

Tri-layered scaffolds also supported HUVSMC attachment, with cells aligning to the PCL fibers, as demonstrated by DAPI-Phalloidin staining (Figure 4F).

### MEW for Fabrication of Patient-Specific Scaffolds

**FIGURE 5:**
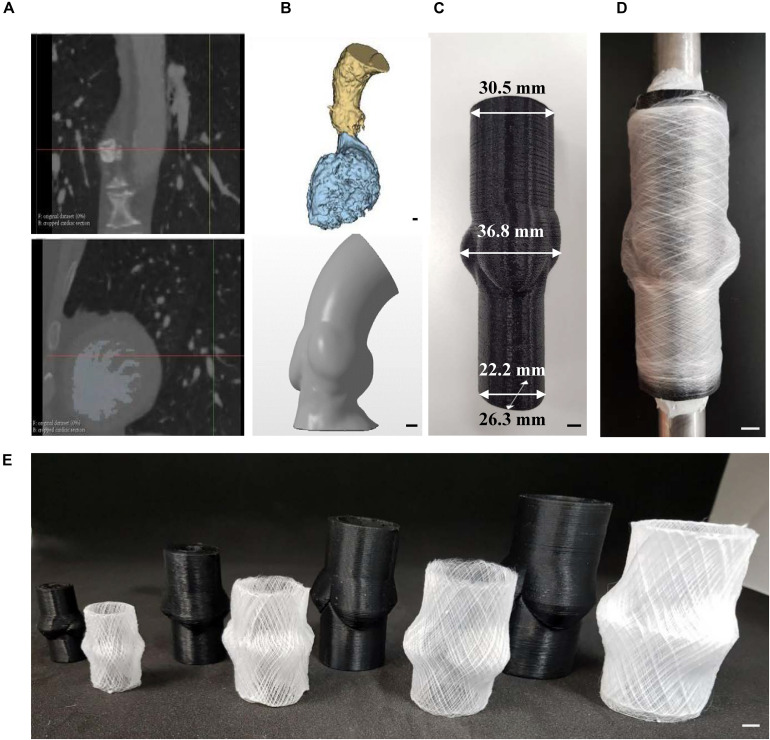
Fabrication of personalized aortic root scaffolds. **(A)** CT scans of the patient’s aorta. **(B)** DICOM to STL conversion using InVesalius software and smooth model of the personalized aorta. **(C)** PLA 3D printed model of the personalized aorta to be used as the substrate mandrel for MEW. **(D)** MEW performed on the 3D printed personalized mandrel. **(E)** 3D printed mandrel models and corresponding MEW scaffolds of 10, 15, 20, and 25 mm diameter. (Scale bar: 5 mm).

Major developments have been undertaken in utilizing patient specific medical devices fabricated through 3D printing for different applications in the medical sector (Limmahakhun et al., 2017; Mohseni et al., 2019). Recently an exciting technology called personalized external aortic root support (PEARS) has emerged as a powerful pre-emptive operation to treat patients suffering from aortic root aneurysm (Treasure et al., 2014, 2016; Izgi et al., 2018). This technique takes advantage from the advancements in medical imaging and 3D printing to fabricate a personalized model of the patient’s aorta, which is then used to fabricated a mesh sleeve that is purposed to restrict aortic root expansion. Given the laborious and time-consuming nature of this method to manually fit these supports to the models, we aimed to utilize the proposed MEW platform to introduce full automation, minimizing the complexity of this process. To highlight the capabilities of MEW to manufacture personalized scaffolds, we fabricated a customized 3D model using a patient-specific geometry obtained from a patient’s aortic root CT scan (Figures 5A–C). This mold was readily incorporated into the versatile MEW platform where scaffolds were fabricated with an exemplary 30° winding angle according to the aforementioned process parameters discussed (Figure 5D). The stability of MEW printing on the personalized mold allowed to manufacture aortic root scaffolds including patient-specific anatomic features (Figure 5C). The ability of this platform to integrate with this exciting treatment approach elucidates the potential of MEW scaffolds for the PEARS procedure. In addition, the scalability of the process was investigated by fabricating hybrid collectors ranging from 10 to 25 mm in diameter. Scaffolds produced on these models demonstrated the capability of this platform to generate scaffolds suitable for patients of all ages (Figure 5E) (Roubertie et al., 2009; Yoganathan et al., 2013). The ability to fabricate anatomically relevant scaffolds at smaller scales has significant implications for the development of tissue engineered heart valves for pediatric patients.

## Conclusion and Future Outlook

In this study, we present a simple, cost-effective and robust method for the manufacture of scaffolds with anatomically relevant 3D geometries and controlled microarchitecture via MEW, here exemplified by the fabrication of biomimetic aortic root scaffolds featuring SV. This significantly expands the capabilities of this technology platform not only for cardiovascular tissue engineering but also for a much wider range of biomedical applications.

## Data Availability Statement

The raw data supporting the conclusions of this article will be made available by the authors, without undue reservation.

## Ethics Statement

The studies involving human participants were reviewed and approved by University Hospital Aachen Local Ethics Committee (EK2067) and Office of Research Ethics and Integrity (OREI) of the Queensland University of Technology (QUT1900000599). The patients/participants provided their written informed consent to participate in this study.

## Author Contributions

NS, ED-J-P, PM, and DH designed the experiments and conceptualized the manuscript. NS conducted the majority of the experimental work, wrote the manuscript, and prepared the figures. ED-J-P and PM reviewed the manuscript. TS performed SEM and Micro CT. DR-G performed the biological evaluation. MM assisted in personalized scaffold design. OB and TH assisted the experimental work. All authors provided feedback. ED-J-P and PM supervised the project, share equal senior contribution, and finalized the manuscript together with NS. All authors approved the final version of the manuscript.

## Conflict of Interest

NS, OB, DH, PM, and ED-J-P declare that they have a pending patent (PCT/AU2020/050383) related to this research. The remaining authors declare that the research was conducted in the absence of any commercial or financial relationships that could be construed as a potential conflict of interest.
