# Supplementary material for: Melt Electrowriting of Complex 3D Anatomically Relevant Scaffolds
Source: Front Bioeng Biotechnol. 2020 Jul 24;8:793. doi: 10.3389/fbioe.2020.00793 (PMC7396698; doi:10.3389/fbioe.2020.00793)
Supplement: Supplementary file 1 [file Data_Sheet_1.pdf]

Melt Electrowriting of Complex 3D Anatomically Relevant Scaffolds

Navid T. Saidy[1,2], Tara Shabab[1], Onur Bas[1,3], Diana M. Rojas-González[4], Matthias Menne[5], Tim Henry[1], Dietmar W. Hutmacher[1,3,6], Petra Mela[4,7*] and Elena M. De-Juan-Pardo[8,9,1*]

[1]Centre in Regenerative Medicine, Institute of Health and Biomedical Innovation (IHBI), Queensland University of Technology (QUT), 60 Musk Avenue, Kelvin Grove 4059, QLD, Australia

[2]The University of Queensland, School of Dentistry, Herston, Queensland, Australia

[3]Department of Biohybrid & Medical Textiles (BioTex), AME-Institute of Applied Medical Engineering, Helmholtz Institute, RWTH Aachen University, Forckenbeckstr. 55, 52074, Aachen, Germany

[4]ARC ITTC in Additive Biomanufacturing, Queensland University of Technology, Musk Avenue, Kelvin Grove, Brisbane, Queensland 4059, Australia

[5]Department of Cardiovascular Engineering, Institute of Applied Medical Engineering, Helmholtz Institute, RWTH Aachen University, Aachen, Germany

[6]Institute for Advanced Study, Technical University of Munich, D-85748 Garching, Germany

[7]Medical Materials and Implants, Department of Mechanical Engineering, Technical University of Munich, Boltzmannstr. 15, 85748 Garching, Germany

[8]Translational 3d Printing Laboratory for Advanced Tissue Engineering (T3mPLATE), Harry Perkins Institute of Medical Research, QEII Medical Centre, Nedlands and Centre for Medical Research, The University of Western Australia, Perth, WA 6009, Australia

[9]School of Engineering, The University of Western Australia, Perth, WA 6009, Australia

[*]Equal contribution and corresponding authors:

Elena M. De-Juan-Pardo: elena.juanpardo@uwa.edu.au
Petra Mela: petra.mela@tum.de

Keywords: Melt electrowriting, 3D printing, Biomimetic, Fused deposition modelling, Personalized scaffolds

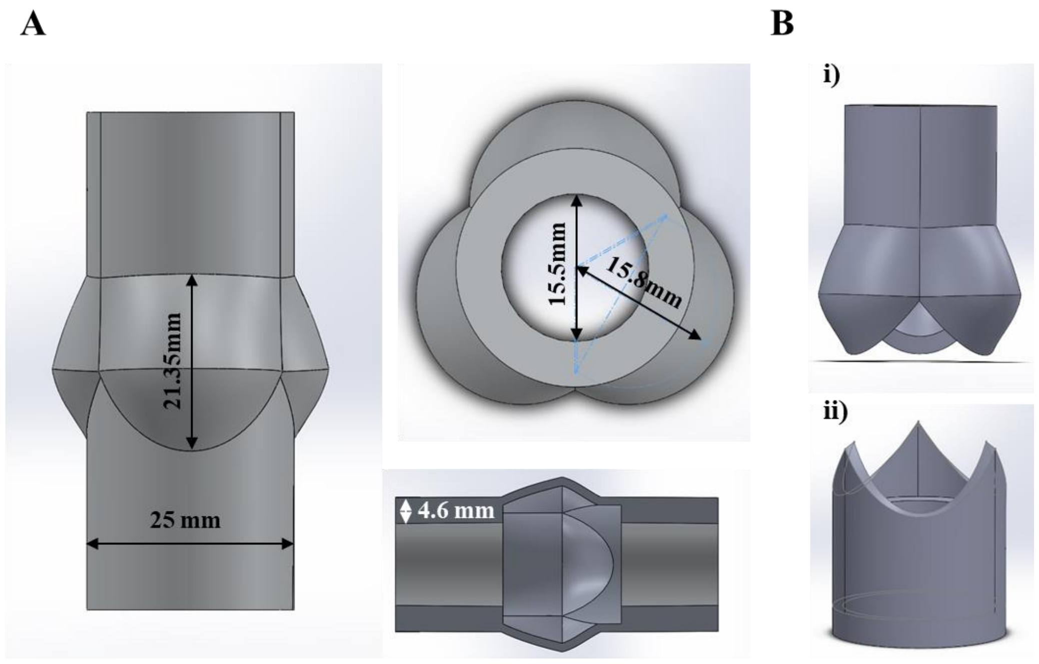

Supplementary Figure S1. 3D model of the aortic root including the sinuses of Valsalva. A) Full model and the details of the dimensions. B) 2-part model designed for better removal of the scaffold from the model.

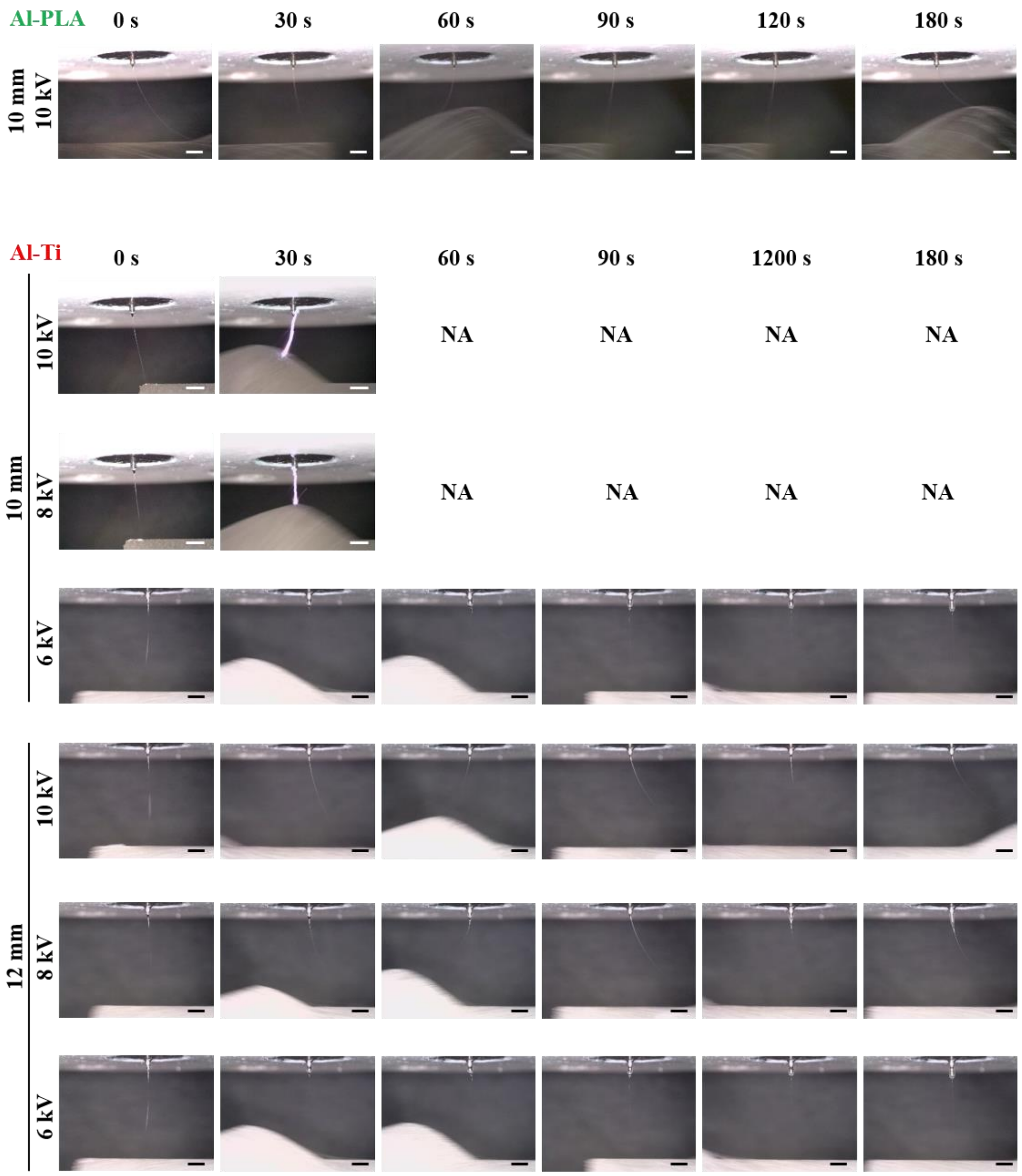

Supplementary Figure S2. Experimental investigation of the influence of mandrel material model and the associated electric field on MEW process: Images of MEW fiber deposition on Al-PLA and Al-Ti mandrels at various voltages and working distances.

A

Tri – Layered Aortic Wall Collagen Fibre Orientation					
Layer	Collagen Fibre Angle	Variable Vector Speed		Constant Vector speed	
		Translational Speed (mm/min)	Rotational Speed (RPM)	Translationa l Speed (mm/min)	Rotational Speed (RPM)
**Intima**	50.0	1000.0	13.1	1812.6	16.7
**Media**	65.0	1000.0	23.6	1000.0	23.6
**Adventitia**	40.0	1000.0	9.2	1521.0	19.9

B

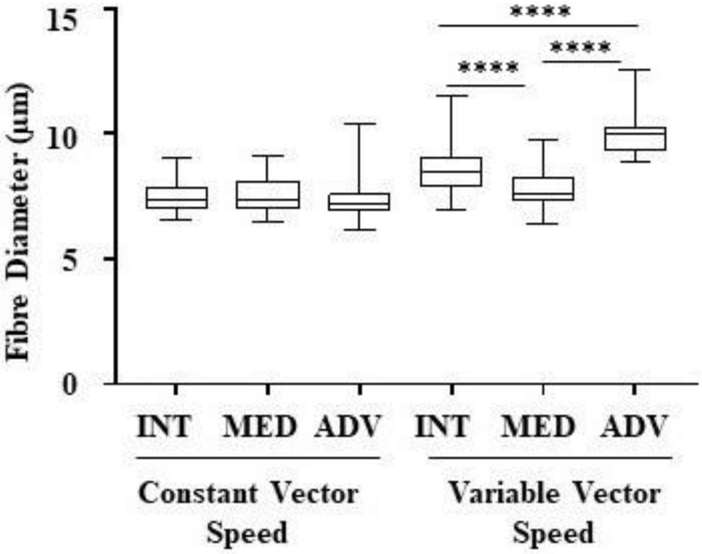

Supplementary Figure S3. A) Collagen fiber angles of the three layers of native aortic roots and the established printing parameters for every layer (Schriefl et al., 2012). B) Scaffolds printed at constant and variable vector speed to maintain fiber diameters.

Supplementary Video 1 *In silico* electric field modelling for MEW on an AL-PLA aortic root model for translational movement

Supplementary Video 2 *In silico* electric field modelling for MEW on an AL-PLA aortic root model for rotational movement

Supplementary Video 3 *In silico* electric field modelling for MEW on an AL-Ti aortic root model for translational movement

Supplementary Video 4 *In silico* electric field modelling for MEW on an AL-Ti aortic root model for rotational movement

Supplementary Video 5 MEW performed on a Ti mandrel at 10 mm working distance and 10 kV

Supplementary Video 6 MEW performed on a Ti mandrel at 10 mm working distance and 8 kV

Supplementary Video 7 MEW performed on a Ti mandrel at 10 mm working distance and 6 kV

Supplementary Video 8 MEW performed on a Ti mandrel at 12 mm working distance and 8 kV

Supplementary Video 9. MEW performed on a Ti mandrel at 12 mm working distance and 6 kV

Supplementary Video 10. MEW performed on a Ti mandrel at 12 mm working distance and 10 kV
